# A Minimal Model Approach for Analyzing Continuous Glucose Monitoring in Type 2 Diabetes

**DOI:** 10.3389/fphys.2018.00673

**Published:** 2018-06-04

**Authors:** Pranay Goel, Durga Parkhi, Amlan Barua, Mita Shah, Saroj Ghaskadbi

**Affiliations:** ^1^Department of Biology, Indian Institute of Science Education and Research, Pune, India; ^2^Indian Institute of Science Education and Research, Pune, India; ^3^Department of Mathematics, Indian Institute of Technology, Dharwad, India; ^4^Global Hospitals, Mumbai, India; ^5^Department of Zoology, Savitribai Phule Pune University, Pune, India

**Keywords:** continuous glucose monitoring, minimal model, type 2 diabetes, insulin estimation, glucose rate of appearance

## Abstract

Continuous glucose monitoring (CGM), a technique that records blood glucose at a regular intervals. While CGM is more commonly used in type 1 diabetes, it is increasingly becoming attractive for treating type 2 diabetic patients. The time series obtained from a CGM provides a rich picture of the glycemic state of the subjects and may help have tighter control on blood sugar by revealing patterns in their physiological responses to food. However, despite its importance, the biophysical understanding of CGM is far from complete. CGM data series is complex not only because it depends on the composition of the food but also varies with individual physiology. All of these make a full modeling of CGM data a difficult task. Here we propose a simple model to explain CGM data in type 2 diabetes. The model combines a relatively simple glucose-insulin dynamics with a two-compartment food model. Using CGM data of a healthy and a diabetic individual we show that this model can capture liquid meals well. The model also allows us to estimate the parameters in a relatively straightforward manner. This opens up the possibility of personalizing the CGM data. The model also predicts insulin time series from the model, and the rate of appearance of glucose due to food. Our methodology thus paves the way for novel analyses of CGM which have not been possible before.

## 1. Introduction

Diabetes is a disease in which glucose is the central measure not only of pathogenesis and diagnosis but also its treatment. Clinically, blood glucose is typically measured as fasting and postprandial plasma glucose, or as glycated hemoglobin. There has been considerable interest in technologies that ease glucose monitoring and improve the resolution of data collection. Continuous glucose monitoring (CGM) uses a sensor, typically fixed on the arm, reports blood glucose every 15 min for a 2 week period. This is a high quality, high time resolution methodology that is becoming increasingly available. The U.S. Food and Drug Administration, for example, has recently approved use of the FreeStyle Libre Flash CGM (FDA News Release – 2017, 2017) sensor. CGM has the potential to help millions of people the world over who struggle chronically with obesity and diabetes (Bode, 2000; Klonoff, 2005; Deiss et al., 2006; Murphy et al., 2008; Juvenile Diabetes Research Foundation Continuous Glucose Monitoring Study Group et al., 2009): It holds the promise of utilizing information contained in the time series to not only gain insight into food habits, and discover (un)healthy dietary patterns but also to determine effective interventions. However, interpreting CGM traces is complex and often subjective, and there are no consensus algorithms to aid the design of appropriate interventions. Techniques aimed at improving the analyses of blood glucose monitoring are an active field of research (McDonnell et al., 2005; Clarke and Kovatchev, 2009; Signal et al., 2013; Kirchsteiger et al., 2016). This paper is a first attempt at describing models that can potentially help us understand CGM time series in type 2 diabetes.

**Figure 1:**
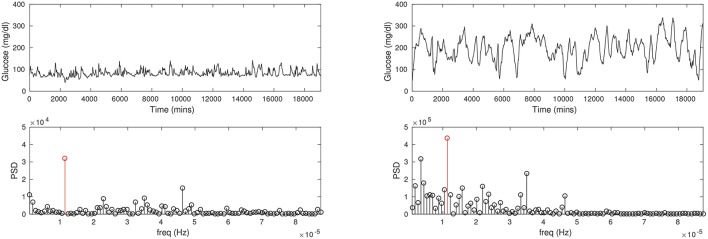
The CGM time series recorded for a healthy, non-diabetic subject (Left column) and a diabetic patient (Right column). Notice the average glucose is substantially lower in the non-diabetic (about 100 mg/dl) than in the diabetic (about 200 mg/dl). The glucose pulses are of large amplitude in the diabetic, and appear to be wider as well. Power spectral density (PSD; second row) shows a prominent peak at a period of about 24 h in either case (red stems), although the frequency profiles are not similar.

At a more fundamental level, CGM reveals differences in the blood glucose *rhythms* of diabetic patients compared to healthy persons, which is considerably more information than just comparisons of the average glucose. Generally speaking, the glucose rhythm in the diabetic can be expected to be more irregular than of a healthy person. Figure 1 shows the CGMs of a healthy subject and a diabetic patient. It is immediately apparent that the mean glucose is considerably higher in the diabetic case. A spectral analysis confirms that there is a sharp peak in the frequency distribution corresponding to a 24-h period (largely due to a periodicity of food intake). While these are straightforward metrics in the time and frequency domains there is considerable additional nuance in these traces, which will be the focus of our attention here. We shall attempt to model the details of the glucose *pulsatility*; this invariably means having to explain the glucose transients that follow meals, breakfast, lunch, dinner, and others. Naively, glucose pulses are driven by food intake, and the restoration of glucose to basal levels (homeostasis) involves the hormone insulin. However, this simplistic fiction hides enormous complexity, and it is fair to say that this process is not yet fully understood. In other words, our model will also encounter this difficulty in one form or another.

In a minimal view of the process, glucose *increase* is driven by hepatic release of blood glucose following food intake, and its *disposal* is driven by (i) insulin-dependent clearance into the peripheral tissue, and (ii) other insulin-independent tissues. The rate of appearance of glucose in the blood following a meal is complicated not only by food composition but also the individual's physiological response to different foods. While glycemic response to a food is correlated to its *glycemic index* (GI), a quantity that expresses how readily it is assimilated and glucose appears in the blood, GI is known to be centered on the ingested *food*, and ignores any person specific physiology. It has been shown that the glycemic response to (the same) food varies considerably between people; interestingly, it appears to depend, in particular, on gut microbiome composition amongst other factors (Zeevi et al., 2015; Korem et al., 2017). A complete, dynamic description of a CGM trace from first principles—that is, starting from a knowledge of foods eaten—appears to be challenging at this time.

On the other hand, there is a considerable body of work centered on modeling glucose and insulin dynamics in type 2 diabetes; for a recent review see (Goel, 2017). One strategy that is often used is to follow the response to a controlled bolus of food. A typical setting is an oral glucose tolerance test (OGTT), in which a very sweet drink (the dose is often 75 grams of glucose) is taken rapidly, and glucose samples are drawn from the blood every 30 min up to 2 h afterwards. Mathematical models involving coupled glucose and insulin dynamics have been used to describe this data in great detail. A significant review of the state-of-the art in this field, in particular of the work of Cobelli and coworkers spanning several decades, appears in Cobelli et al. (2009). We note that while modeling OGTT, and to a lesser extent “mixed-meal” data, are quite mature, to the best of our knowledge a model suitable for CGM has not yet been described. There are several considerations that seem to argue against simply extending one of the earlier models to CGM. The current models involve a number of compartments, not only for glucose and insulin but also liver, muscle and adipose, the gastro-intestinal tract, and so on. As such, it is difficult to envisage the numerous model parameters can all be identified from CGM data alone. In the models described in Cobelli et al. (2009), for example, parameters were discovered using glucose tolerance tests together with various other techniques, such as radio-labeled tracers. Not only are these difficult and expensive experiments in themselves but reproducing these outside the scope of specialized studies is impractical.

Our interest in this paper is to describe a minimal mathematical model useful for exploring CGM data. We restrict ourselves largely to CGM data collection; however, we note that it is plausible to add a few other commonplace measurements including (i) the use of fingerstick glucose measurement, and (ii) a professional (laboratory-based) fasting and postprandial glucose, glycated hemoglobin and insulin measurements carried out a few times while the CGM sensor is implanted. This additional data can help in determining the model, as we describe below. While it is difficult to expect to be able to describe the glucose transient following each meal, we show that liquid meals are relatively easy to describe; this insight will be used to fit model parameters. In other words, we propose a novel strategy for the model personalization of individual CGM data. Our methodology also helps us recover the *insulin* time series corresponding to the CGM, using the fitted model. Further, it is also possible to estimate the time series corresponding to the appearance of glucose due to food. Finally, we estimate clinically important parameters corresponding to the insulin secretion capacity of the pancreas and insulin resistance, and compare them between the normal and diabetic cases. Our methodology appears to be suitable for CGM users widely, with few additional measurements required.

## 2. Models and methods

### 2.1. Data collection

The digital CGM records of two individuals, a diabetic patient undergoing treatment and a healthy subject, were analyzed retrospectively. Note that the patient is not in a controlled environment, they carry out their daily activities independently with the sensor attached to them. The CGM sensor is about the size of a coin, and is typically affixed to the arm. The patient is fully ambulatory and only minimally aware of the sensor, that is, it does not interfere with most routine activities. The sensor collects data continuously for about 2 weeks.

All subjects gave written informed consent for use of their data. A separate approval from the Institutional Ethics Committee of Global Hospitals was not requested since data from only two individuals was analyzed here, and this does not represent a full scale human clinical study. In particular, diabetes treatment and CGM monitoring is at the discretion of the doctor and patient, and data was only analyzed after data collection.

### 2.2. A minimal model of glucose and insulin dynamics

We adapt a glucose–insulin model due to (Topp et al., 2000), and add food dynamics to it as follows.

#### 2.2.1. Food dynamics

The digestion of food is modeled using two-compartment dynamics. Food enters the first compartment, the stomach, *q*_*sto*_, and is passed along to a second compartment, collectively called the “gut”, *q*_*gut*_, from which glucose is assimilated into the bloodstream. Thus,

(1) $$\begin{array}{r} \frac{d{\;q}_{sto}}{dt}=-k_{sto}q_{sto}, \end{array}$$

(2) $$\begin{array}{r} \frac{dq_{gut}}{dt}=k_{sto}q_{sto}-k_{gut}q_{gut}, \end{array}$$

where *q*_*sto*_ ← *q*_*sto*_ + *food*_*i*_ is the food intake at times *t*_*i*_. We demonstrate below that this is a good model that captures the glucose response to liquid foods. Modeling mixed-meals is considerably more complex and is not carried out here, however, we show that an average description can sometimes be achieved by modeling such meals with the two-compartment model above, except with a different value of *k*_*gut*_. That is, for liquid meals we take *k*_*gut*_ ≡ *k*_*gut, l*_ while for a mixed-meal we set *k*_*gut*_ ≡ *k*_*gut, mm*_.

#### 2.2.2. The topp model of glucose–insulin dynamics

The *G* − *I* model for the dynamics of glucose and insulin is:

(3) $$\begin{array}{rc} \frac{dG}{dt} & =R_{0}-\left(EG_{0}+S_{I}I\right)G+k_{gut}{\;q}_{gut}, \end{array}$$

(4) $$\begin{array}{rc} \frac{dI}{dt} & =I_{max}\frac{G^{2}}{\alpha +G^{2}}-k_{I}I, \end{array}$$

where glucose absorption occurs from *q*_*gut*_. *R*_0_ stands for a basal production of glucose and *EG*_0_ is insulin-independent glucose utilization, *S*_*I*_ is insulin sensitivity and determines the insulin-dependent glucose clearance from the blood. *I*_*max*_ is the maximal rate of insulin secretion from the pancreas and *k*_*I*_ the rate at which insulin is cleared (largely by the liver). *G* is measured in mg/dl, and *I* in μU/ml.

### 2.3. Model fitting

We fit parameters of the model following a standard optimization approach. The cost function we optimize typically involves the CGM time series around the pulse that we wish to focus on fitting.

The simulation of the model ODEs is carried out as follows. At time *t* = *t*_0_, there is no food in the system and we allow the model to evolve; for each food that enters the system, *q*_*sto*_ is adjusted to a value *q*_*sto*_ + *food*_*i*_ where *food*_*i*_ is the spike in the value of *q*_*sto*_ at time *t*_*i*_. The *food*_*i*_ are a part of the optimization problem. The optimization algorithm then identifies the model parameters and food sizes; the cost function minimized is a squared difference between experimental values of CGM and simulation.

Numerical experiments indicate that the parameters *EG*_0_ and α are not identifiable; these values are kept fixed (see Table 1 for the values) close to the nominal values used in Topp et al. (2000). The steady state of insulin can be obtained from Equation (4) as $\frac{I_{max}G_{ss}^{2}}{\alpha +G_{ss}^{2}}/k_{I}$; since we assume we know the fasting insulin from a laboratory measurement in our method, we allow *k*_*I*_ to be determined by the above, namely, $k_{I}\equiv \frac{I_{max}G_{ss}^{2}}{\alpha +G_{ss}^{2}}/I_{ss}$. For the non-diabetic case we use *G*_*ss*_ = 90 and *I*_*ss*_ = 12.4, and for the diabetic case *G*_*ss*_ = 195 and *I*_*ss*_ = 12.5.

**Table 1:** Parameters corresponding to the non-diabetic and diabetic CGM time series.

**Parameter**	**Non-diabetic**	**Diabetic**	**Units**
*R*_0_	2.1	2.5	mgdl^−1^min^−1^
*E*_*G*0_	1 × 10^−3^	2.5 × 10^−3^	min^−1^
*S*_*I*_	3.06 × 10^−3^	1.14 × 10^−3^	ml μU^−1^min^−1^
α	1 × 10^4^	1 × 10^4^	mg^2^dl^−2^
*I*_*max*_	0.28	0.93	μU ml^−1^min^−1^
*k*_*I*_	0.01	0.06	min^−1^
*k*_*sto*_	0.036	0.026	min^−1^
*k*_*gut, l*_	0.098	0.026	min^−1^
*k*_*gut, mm*_	0.011	–	min^−1^

The model simulations and optimization are carried out in MATLAB. ode45 is used for ODE integration, while fitting uses a combination of patternsearch and fmincon with suitable constraints. Optimzation iterations were terminated when the relative changes in all elements of the parameter vector were lower than a nominal tolerance of 10^−6^.

## 3. Results

### 3.1. CGM of a non-diabetic subject

**Figure 2:**
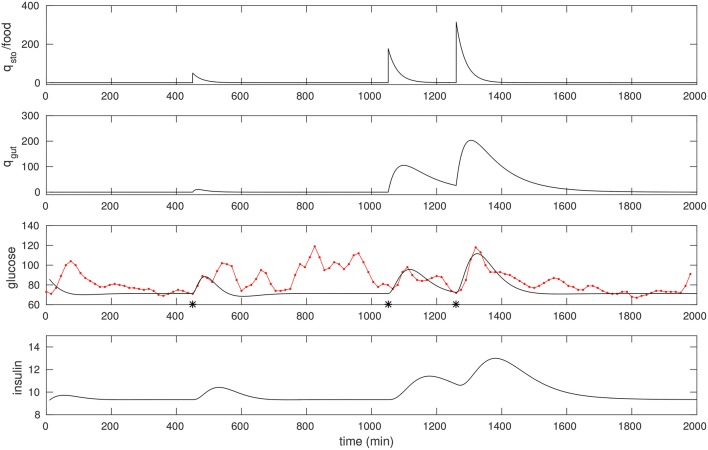
The CGM of around 24 h of a non-diabetic subject, starting at midnight. The glucose data (red) is overlaid with the model simulation (black). Three foods were selected, and the times they were taken (450, 1,051, and 1,260 respectively) were recalled from the food diary; these are marked with asterisks. The first is a liquid meal at breakfast while the other two are mixed meals (the last one being dinner). These food pulses were fit; the fitted *q*_*sto*_ amplitudes are 50, 177, and 316 respectively.

We found that a few “landmark” pulses are sufficient to estimate the *G*−*I* model. In Figure 2 we fit three peaks that had a significant liquid component to it. The resulting model parameters are shown in Table 1.

The following quick checks indicate this is a reasonable fit: The relative sizes of food estimates, the three *q*_*sto*_ pulses, are commensurate with the corresponding glucose peaks, and with the diet diary. The insulin response is peaked similarly, as expected. In particular, the physiological range of insulin is typically between 2 and 25 μU/ml, consistent with the result here. Insulin peaks occur slightly later than the glucose peaks. Finally, note that the response to liquid meal at 450 min is fit well (this is by design: That pulse is weighted relatively heavily in the cost function) while the other two responses to mixed meals are more complex (especially the postprandial clearance), and these are fit in an “average” sense, in line with expectation.

Note the resting glucose and insulin obtained through the fitting process. The resting insulin is approximately 9 μU/ml, slightly lower than the laboratory fasting insulin measurement, 12.5. This appears to be consistent with the observation that resting glucose over this epoch is close to 70 mg/dl.

These fits suggest a straightforward procedure for obtaining a personalized model of CGM, that is, for fitting model parameters to describe a particular time series. An “isolated” liquid meal – that is, taken with a sufficient gap before and after it – is described well by the present model, and one readily recovers all parameters through optimization.

### 3.2. CGM of a diabetic patient

**Figure 3:**
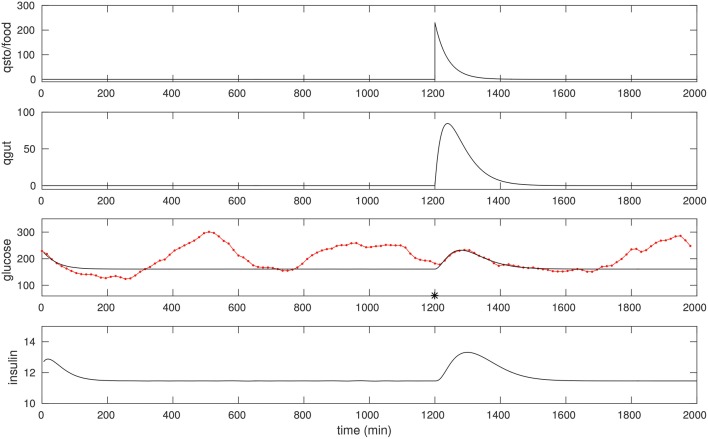
The CGM of about 24 h of a diabetic subject, starting at around 0400 h. The glucose data (red) is overlaid with the model simulation (black). An isolated (liquid) food pulse at 1,200 min in this trace has been fit. *EG*_0_ was set to 2.5 × 10^−3^, the other parameters were estimated as in the non-diabetic case; see text for details. The fitted *q*_*sto*_ amplitude is 230.

We optimize over a suitable peak in the CGM of a diabetic patient in a manner similar to that of the non-diabetic case. Most meals for this individual were of mixed-meal type, however, we noted a few that were liquid meals. One such peak we believe was due to a liquids taken around 1,200 min, see Figure 3. Once again, fitting this peak allows us to recover suitable model parameters; these are listed in Table 1. One feature to note is that the *EG*_0_ appears to be larger for this individual (this can be estimated by fitting a later night portion of the CGM, when insulin is not likely to be dominant, with an exponential).

### 3.3. Insight into diabetogenesis

A comparison of the parameters between the non-diabetic and diabetic cases is instructive. We note, first, that our procedure seems to be robust in determining parameters. In particular, we obtain the clinically important parameters, *S*_*I*_ and *I*_*max*_ among other things. We generally expect that insulin sensitivity ought to be lowered in diabetes. Maximal secretion typically first rises as diabetes develops (to account for the increasing demand that hyperglycemia places on it), and as exhaustion sets in, secretion deteriorates. From Table 1 it is seen that the estimated *I*_*max*_ is larger for the diabetic patient; this can either be because insulin is in the compensatory phase of diabetogenesis, or as is more likely, the result of secretagogue drugs prescribed to them. *S*_*I*_, on the other hand, is lower for the diabetic person as expected.

### 3.4. Estimation of insulin

An immediate application of personalizing the model fits is to estimate insulin. Insulin is not easy to measure clinically as it is expensive and requires drawing blood. Furthermore, no technology exists currently for continuous *insulin* monitoring. Using the model fit it is possible to estimate insulin with the same time resolution as CGM. We show this next.

We design an observer (Moreno, 2000; Robenack and Goel, 2007), Î, to estimate the insulin dynamics, $\frac{dI}{dt}=I_{\infty }\left(G\right)-k_{I}I$, Equation 4. We note that the equation

(5) $$\begin{array}{r} \frac{dÎ}{dt}=I_{\infty }\left(G_{data}\right)-k_{I}Î, \end{array}$$

driven by *G*_*data*_, the CGM time series, converges exponentially to the true dynamics of the system, that is, Î → *I*, since the error, *e* ≡ *I* − Î satisfies ė = −*k*_*I*_*e*. In other words, apart from an initial transient that corresponds to the time constant with which the error dissipates, 1/*k*_*I*_, Î reports the times series *I*.

**Figure 4:**
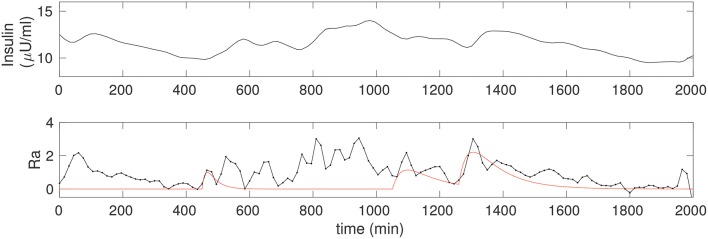
**(Upper)** The insulin time series corresponding to the non-diabetic glucose from Figure 2, estimated from the fitted model. Notice that, apart from an initial transient, the insulin is estimated throughout, and this does not require any knowledge of the food input. **(Lower)** The estimate of the rate of appearance of glucose due to food, *Ra* (black). The simulation of *k*_*gut*_*q*_*gut*_ from Figure 2 is overlaid (red) for comparison.

Figure 4 shows the insulin estimated for the non-diabetic case. Insulin is obtained continuously in time, and since it requires only the glucose data it can be computed even without a detailed knowledge of the food intake. Notice that the insulin in Figure 2 is computed for the glucose corresponding to the three food peaks, whereas the insulin in Figure 4 is computed using the observer above from the CGM data, and is a faithful representation of its co-variation with glucose; in particular, Î can be seen to be considerably more nuanced.

Apart from being a very useful facility in general, this technique can be particularly valuable in a setting where one wishes to know the insulin response to a certain food. There are theories of obesity [the “carbohydrate–insulin model”; (see, for example, Ludwig and Friedman, 2014; Goel, 2017)] as well as diabetes (Corkey, 2012; Goel, 2015) that rest on the insulin response to food. It would be interesting to evaluate the techniques here in the context of such studies.

### 3.5. Estimation of the rate of appearance of glucose

It is interesting to know what is the rate of appearance of glucose, *Ra*, due to food. That is, in Equation 3, $\frac{dG}{dt}=g\left(G,I\right)+Ra$, where *g*(*G, I*) = *R*_0_ − (*EG*_0_ + *S*_*I*_*I*)*G* and *Ra* is modeled as *k*_*gut*_*q*_*gut*_. However, we would like to solve an inverse problem to directly determine what the *Ra* is more generally. In other words, given that we have estimated the model parameters, we would like to design an input observer for *Ra*. An estimate of *Ra* can be computed as

(6) $$\begin{array}{r} Ra=\frac{dG_{data}}{dt}-R_{0}+\left(EG_{0}+S_{I}Î\right)G_{data}, \end{array}$$

where $\frac{dG_{data}}{dt}$ is computed directly from differentiating the CGM time series, and insulin is estimated via Î, Equation 5. The observed *Ra* is shown in Figure 4, lower. A spline fit was used to determine the required derivative in order to reduce numerical inaccuracy. For comparison, we have overlaid the solution from Figure 2, that is, *Ra* computed from the input observer is compared to the case with three food pulses, modeled as *Ra* ≡ *k*_*gut*_*q*_*gut*_. The correspondence between the two is excellent for the fully liquid meal, and reasonable for the other two as well.

The input observer for *Ra* thus gives us an excellent facility not only to discover the effect of different foods on the appearance of glucose in the blood but also for carrying out modeling studies to try and explain mixed meals. This will be investigated in greater detail in future work.

## 4. Discussion

In our view CGM is a very powerful data collection tool in diabetes. Despite its importance, models do not yet exist for describing it. Here we show that parts, if not all, of the data can be explained rather simply, and more importantly, one can recover all of the model parameters required to fit CGM time series individually. Our key observation is that liquids seem to pass through the gut in a fairly stereotyped manner, and we are able to model this successfully. We demonstrate that the glycemic response to a liquid meal, especially one that is well separated from other food intakes, can be used to fit a minimal mathematical model, and identify all the parameters needed to describe glucose and insulin dynamics well. Thus, our prescription for fitting a personalized model to a CGM time series is simply ask the patient to take a liquid meal by itself. In some respects this is similar to an OGTT test, however, there are differences: For one, our method does not require the meal to be in glucose or sucrose form, and it is not necessary to know the quantity a priori; the algorithm determines it anyway. This makes our method flexible, and practical. Finally, we recover the clinically important indices of insulin resistance and secretory capacity that have been traditionally used to describe the progression, and state of the disease. We are also able to use the model to estimate a corresponding insulin time series, as well as the rate of appearance of glucose due to food.

Our model can be refined further if additional measurements can be taken. In particular, more insulin measurements taken during the CGM period can substantially improve the fits, especially of insulin.

It is straightforward to record food timings in a diary, and this very useful to the model fitting. We note that the time at which the food intake is *started* was more important than when it was completed. Complex food models can presumably use more information. More generally, it is of great interest to explain the glycemic response to different foods, and while the observer we have constructed for *R*_*a*_ greatly helps facilitate this, we have not offered any particular direction in that regard in this paper. Our preliminary attempts at using Type I mixed-meal models (see Li et al., 2016 for details), to explain that data in Figure 2 for example, indicate these models do not appear to be satisfactory. We hope to examine this further in future work.

One weakness of our study is that hypoglycemic events, in general, are not captured well by this model. This is because the model focuses on glucose and insulin dynamics, that is, on postprandial events when glucose is elevated. That our simple model is unable to capture hypoglycemia is not too surprising, considering the complexity associated with understanding this phenomena in general (American Diabetes Association Workgroup on Hypoglycemia, 2005; Unger, 2012; Elliott et al., 2016). A substantial body of literature exists trying to explain it in different contexts such as in juvenile diabetes (Juvenile Diabetes Research Foundation Continuous Glucose Monitoring Study Group et al., 2011), or for type I diabetes patients (Kim et al., 2011), and in assessing its impact on productivity (Brod et al., 2011). Notably, Sampath et al. (2016) have recently proposed a machine learning algorithm that combines different glycemic indices to successfully predict occurrences of nocturnal hypoglycemic incidents. In order to model hypoglycemic events more carefully the model should probably be extended to include glucagon dynamics as well; these questions will be explored further in future work.

There have been concerns raised previously that because CGM sensors measure interstitial glucose, that is, in a remote compartment just below the skin, this may be different from blood glucose that is measured in a fingerstick or laboratory testing. A number of studies have been carried out to model a relationship between the two (see for example Cobelli et al., 2009). In addition, there appears to be a small time lag (about 5 min) between the CGM and blood glucose. On the other hand, it is now widely accepted that CGM readings do not require confirmation (calibration) against a laboratory (or fingerstick) sampling (FDA News Release – 2017, 2017). For instance, the mean absolute relative difference (MARD) between the Freestyle Libre sensor reading and capillary blood glucose (BG) has previously been reported to be about 11.4% overall (Bailey et al., 2015). Our own tests comparing random samples (RBS) of blood glucose (laboratory testing) to the sensor indicate a strong correlation, *G*_*CGM*_ = 0.96*RGB*−14 (*n* = 35, *R* = 0.97; data not shown). In this study we have ignored any calibration considerations between blood glucose and sensor readings, and any time lag; we have tacitly assumed laboratory and sensor readings and are directly compatible. It will be of interest to establish more carefully how to adapt the model to explain such differences, if any. More generally, uncertainty quantification for our model will be carried out elsewhere.

Despite the numerous limitations of understanding CGM data in all its complexity, we have shown that a minimal model is readily identifiable from the time series. We have thus provided a proof of concept that our methodology appears to be viable strategy toward a personalized analysis of CGM. This holds tremendous potential for various kinds of investigations, not only to recommend diet and lifestyle interventions but also test the exact effect of drugs. While such details are beyond the scope of the present paper, we hope the results presented here pave the way for further research in this direction.

## Author contributions

PG, MS, and SG designed the study. PG designed the analysis. PG, DP, and AB carried out the simulations. PG and AB wrote the paper, together with MS and SG.

### Conflict of interest statement

The authors declare that the research was conducted in the absence of any commercial or financial relationships that could be construed as a potential conflict of interest. The reviewer ABP and handling Editor declared their shared affiliation.

## References

[B1] American Diabetes Association Workgroup on Hypoglycemia (2005). Defining and reporting hypoglycemia in diabetes: a report from the American Diabetes Association Workgroup on Hypoglycemia. Diab. Care 28, 1245–1249. 10.2337/diacare.28.5.1245 15855602

[B2] Bailey T. Bode B. W. Christiansen M. P. Klaff L. J. Alva S. (2015). The performance and usability of a factory-calibrated flash glucose monitoring system. Diab. Technol. Ther. 17, 787–794. 10.1089/dia.2014.0378 26171659 PMC4649725

[B3] Bode B. W. (2000). Clinical utility of the continuous glucose monitoring system. Diab. Technol. Therapeut. 2(Suppl. 1), 35–41. 10.1089/15209150050214104 11469630

[B4] Brod M. Christensen T. Thomsen T. L. Bushnell D. M. (2011). The impact of non-severe hypoglycemic events on work productivity and diabetes management. Value Health 14, 665–671. 10.1016/j.jval.2011.02.001 21839404

[B5] Clarke W. Kovatchev B. (2009). Statistical tools to analyze continuous glucose monitor data. Diab. Technol. Therap. 11, S-45–S-54. 10.1089/dia.2008.0138 19469677 PMC2903980

[B6] Cobelli C. Man C. D. Sparacino G. Magni L. De Nicolao G. Kovatchev B. P. (2009). Diabetes: models, signals, and control. IEEE Rev. Biomed. Eng. 2, 54–96. 10.1109/RBME.2009.2036073 20936056 PMC2951686

[B7] Corkey B. E. (2012). Diabetes: have we got it all wrong? Insulin hypersecretion and food additives: cause of obesity and diabetes? Diab. Care 35, 2432–2437. 10.2337/dc12-0825 23173132 PMC3507569

[B8] Deiss D. Bolinder J. Riveline J. P. Battelino T. Bosi E. Tubiana-Rufi N.. (2006). Improved glycemic control in poorly controlled patients with type 1 diabetes using real-time continuous glucose monitoring. Diab. Care 29, 2730–2732. 10.2337/dc06-1134 17130215

[B9] Elliott L. Fidler C. Ditchfield A. Stissing T. (2016). Hypoglycemia event rates: a comparison between real-world data and randomized controlled trial populations in insulin-treated diabetes. Diab. Ther. 7, 45–60. 10.1007/s13300-016-0157-z 26886441 PMC4801820

[B10] FDA News Release – 2017 (2017). FDA Approves First Continuous Glucose Monitoring System for Adults Not Requiring Blood Sample Calibration. Available online at: https://www.fda.gov/NewsEvents/Newsroom/PressAnnouncements/ucm577890.htm

[B11] Goel P. (2015). Insulin resistance or hypersecretion? The βIG picture revisited. J. Theor. Biol. 384, 131–139. 10.1016/j.jtbi.2015.07.033 26300065

[B12] Goel P. (2017). Chapter 13 - theoretical advances in type 2 diabetes, in Disease Modelling and Public Health, Part A, Vol. 36 of Handbook of Statistics, eds Rao A. S. S. Pyne S. Rao C. (Amsterdam: Elsevier), 369–395.

[B13] Juvenile Diabetes Research Foundation Continuous Glucose Monitoring Study Group Beck R. W. Hirsch I. B. Laffel L. Tamborlane W. V. Bode B. W.. (2009). The effect of continuous glucose monitoring in well-controlled type 1 diabetes. Diab. Care 32, 1378–1383. 10.2337/dc09-0108 19429875 PMC2713649

[B14] Juvenile Diabetes Research Foundation Continuous Glucose Monitoring Study Group Fiallo-Scharer, R. Cheng J. Beck R. W. Buckingham B. A. Chase H. P. (2011). Factors predictive of severe hypoglycemia in type 1 diabetes. Diab. Care 34, 586–590. 10.2337/dc10-1111 PMC3041185 21266651

[B15] Kim S. K. Suh S. Kim M. Y. Chung H. S. Hur K. Y. Kim S. W.. (2011). Three-day continuous glucose monitoring for rapid assessment of hypoglycemic events and glycemic variability in type 1 diabetic patients. Endocrine J. 58, 535–541. 10.1507/endocrj.K10E-378 21532214

[B16] Kirchsteiger H. Jorgensen J. Renard E. del Re L. (eds.). (2016). Prediction Methods for Blood Glucose Concentration. Design, Use and Evaluation. Lecture Notes in Bioengineering, Vol. 7 New York, NY: Springer.

[B17] Klonoff D. C. (2005). Continuous glucose monitoring. Diab. Care 28, 1231–1239. 10.2337/diacare.28.5.1231 15855600

[B18] Korem T. Zeevi D. Zmora N. Weissbrod O. Bar N. Lotan-Pompan M.. (2017). Bread affects clinical parameters and induces gut microbiome-associated personal glycemic responses. Cell Metab. 25, 1243–1253. 10.1016/j.cmet.2017.05.002 28591632

[B19] Li Y. Chow C. C. Courville A. B. Sumner A. E. Periwal V. (2016). Modeling glucose and free fatty acid kinetics in glucose and meal tolerance test. Theor. Biol. Med. Model. 13:8. 10.1186/s12976-016-0036-3 26934990 PMC4776401

[B20] Ludwig D. S. Friedman M. I. (2014). Increasing adiposity: consequence or cause of overeating? JAMA 311, 2167–2168. 10.1001/jama.2014.4133 24839118

[B21] McDonnell C. M. Donath S. M. Vidmar S. I. Werther G. A. Cameron F. J. (2005). A novel approach to continuous glucose analysis utilizing glycemic variation. Diab. Technol. Therapeut. 7, 253–263. 10.1089/dia.2005.7.253 15857227

[B22] Moreno J. (2000). Unknown input observers for siso nonlinear systems, in 39th Conference on Decision and Control, Vol. 1 (New York, NY), 790–801. 10.1109/CDC.2000.912865

[B23] Murphy H. R. Rayman G. Lewis K. Kelly S. Johal B. Duffield K.. (2008). Effectiveness of continuous glucose monitoring in pregnant women with diabetes: randomised clinical trial. BMJ 337:a1680. 10.1136/bmj.a1680 18818254 PMC2563261

[B24] Robenack K. Goel P. (2007). Observer based measurement of the input current of a neuron. Medit. J. Meas. Control 3, 22–29.

[B25] Sampath S. Tkachenko P. Renard E. Pereverzev S. V. (2016). Glycemic control indices and their aggregation in the prediction of nocturnal hypoglycemia from intermittent blood glucose measurements. J. Diab. Sci. Technol. 10, 1245–1250. 10.1177/1932296816670400 27660190 PMC5094347

[B26] Signal M. Thomas F. Shaw G. M. Chase J. G. (2013). Complexity of continuous glucose monitoring data in critically ill patients: continuous glucose monitoring devices, sensor locations, and detrended fluctuation analysis methods. J. Diab. Sci. Technol. 7, 1492–1506. 10.1177/193229681300700609 24351175 PMC3876327

[B27] Topp B. Promislow K. deVries G. Miura R. M. Finegood D. T. (2000). A model of beta-cell mass, insulin, and glucose kinetics: pathways to diabetes. J. Theor. Biol. 206, 605–619. 10.1006/jtbi.2000.2150 11013117

[B28] Unger J. (2012). Uncovering undetected hypoglycemic events. Diab. Metab. Syndr. Obes. Targets Ther. 5:57. 10.2147/DMSO.S29367 22563248 PMC3340111

[B29] Zeevi D. Korem T. Zmora N. Israeli D. Rothschild D. Weinberger A.. (2015). Personalized nutrition by prediction of glycemic responses. Cell 163, 1079–1094. 10.1016/j.cell.2015.11.001 26590418

